# Chromosome-level genome assembly of *Hippophae rhamnoides* variety

**DOI:** 10.1038/s41597-024-03549-w

**Published:** 2024-07-13

**Authors:** Xingyu Yang, Shujie Luo, Shihai Yang, Ciren Duoji, Qianwen Wang, Zhiyu Chen, Danni Yang, Tianyu Yang, Xi Wan, Yunqiang Yang, Tianmeng Liu, Yongping Yang

**Affiliations:** 1 Xishuangbanna Tropical Botanical Garden, Chinese Academy of Sciences, kunming, 650000 China; 2 University of Chinese Academy of Sciences, Beijing, 100049 China; 3 Dali University, Dali, 671000 China; 4 Yunwang Industrial Corporation, Ltd, Tibet, 850000 China; 5 Service Center for Forestry and Grassland Bureau of Sangzhuzi District in Xizang, Xizang, 850000 China; 6 Plant Germplasm and Genomics Center, Kunming Institute of Botany, Chinese Academy of Sciences, Kunming, 650201 China; 7 Institute of Tibetan Plateau Research at Kunming, Kunming Institute of Botany, Chinese Academy of Sciences, Kunming, 650201 China

**Keywords:** Plant evolution, Plant molecular biology

## Abstract

*Fructus hippophae (Hippophae rhamnoides spp. mongolica*×*Hippophae rhamnoides sinensis)*, a hybrid variety of sea buckthorn that *Hippophae rhamnoides* spp. *mongolica* serves as the female parent and *Hippophae rhamnoides* *sinensis* serves as the male parent, is a traditional plant with great potentials of economic and medical values. Herein, we gained a chromosome-level genome of *Fructus hippophae* about 918.59 Mb, with the scaffolds N50 reaching 83.65 Mb. Then, we anchored 440 contigs with 97.17% of the total genome sequences onto 12 pseudochromosomes. Next, *de-novo*, homology and transcriptome assembly strategies were adopted for gene structure prediction. This predicted 36475 protein-coding genes, of which 36226 genes could be functionally annotated. Simultaneously, various strategies were used for quality assessment, both the complete BUSCO value (98.80%) and the mapping rate indicated the high assembly quality. Repetitive elements, which occupied 63.68% of the genome, and 1483600 bp of non-coding RNA were annotated. Here, we provide genomic information on female plants of a popular variety, which can provide data for pan-genomic construction of sea buckthorn and for the resolution of the mechanism of sex differentiation.

## Background & Summary

Sea buckthorn (*Hippophae*), belonging to the Elaeagnaceae family, is a diploid (2n = 2x = 24) deciduous plant with high exploitation values^1,2^. Most sea buckthorn is cultivated in cold zones of Europe and Asia^3,4^. *Hippophae* is rich in ascorbic acid, carotenoids, healthy fatty acids, and other secondary metabolites^5–7^. Previous studies have primarily focused on its medicinal value. Extracts from the leaves and orange-yellow fruit have immunomodulatory potential and antioxidant, anti-viral, and wound-healing properties^8–11^. Sea buckthorn is also used in traditional medicine for the treatment of pulmonary, cardiac, gastrointestinal, blood, or metabolic disorders^12–16^. It is therefore crucial to decode the genomic information of Sea buckthorn. Three genome of *Hippophae* were published last year, including *Hippophae rhamnoides ssp. sinensis*, *Hippophae tibetana*, and *Hippophae gyantsensis* which revealing differences in their biological data, such as the genome size and percentage of repeated sequences^17–19^. The decoding of further genomic information from other *Hippophae* subspecies and popular varieties is therefore of importance.

Rapid advances in sequencing technology have made it possible to obtain accurate and high-throughput data at a very low cost^20,21^. However, there is currently no research on *Fructus hippophae* genomic information. Studies on *Fructus hippophae* are currently limited to compounds and their related protein targets of Hippophae Fructus oil (HFO), relying on the Traditional Chinese Medicine Systems Pharmacology Database and Analysis Platform (TCMSP: https://old.tcmsp-e.com/tcmsp.php)^22^. Other studies have focused on methods of extracting and purifying flavonoids, tannins, and other novel nutritional supplements from *Fructus hippophae*, which depend on spectrophotometry, chromatography and other chemical methods^23–25^. Herein, we integrated three different sequencing datasets for genome assembly, including short reads based on next generation sequencing (NGS) on the MGI platform, Oxford Nanopore Technologies (ONT) long reads, and high-throughput chromatin conformation capture (Hi-C) reads. Structural annotation of protein-coding genes was then carried out by *de novo*, homology and transcriptome assembly strategies. Next, gene functional annotation was performed by alignment with public databases. These genome-related data will provide a valuable resource for the study of sea buckthorn.

## Methods

### Plant materials and genome sequencing

To study the genome of *Fructus hippophae*, fresh young leaves were collected from the same wild *Fructus hippophae* tree which planted in Shigatse, Tibet, China. Total genomic DNA and RNA were extracted using the modified cetyl trimethylammonium bromide (CTAB) method and E.Z.N.A. Total RNA Kit I (Omega Bio-Tek, Norcross, GA, USA), respectively^26^. Then, 150 bp paired-end libraries with an insert size of 250 bp were constructed and sequenced at the MGISEQ-T7 platform. The Hi-C sequencing library was constructed according to the published protocol, and then the crosslinked chromatin was digested with DpnII and ligated after biotinylation. DNA fragments were enriched via the interaction between biotin and blunt-end ligation, and then the enriched library was sequenced on the MGISEQ-T7 platform. DNA Long reads were generated by the Nanopore platform and processed by IsoSeq technology using the SMRT method. A totals of 56 Gb of raw MGI short-read data (61× coverage) with a Q30 exceeding 90% (Table 1), 98 Gb of passed Nanopore long-reads data (93× coverage, the N50 length reaching 36264 bp, the average length reaching 25977 bp) (Table 2), and 79.09 Gb of Hi-C data (86× coverage) with the Q30 reaching 94.23% (Table 1) finally were obtained from the whole-genome sequencing.

**Table 1:** Characteristics of NGS data for genome assembly.

Sequence	Platform	Total bases	GC content (%)	Q30 (%)	Sequence depth (×)
DNA reads	MGISEQ-T7	56050695900	30.30	90.32	61
RNA reads	MGISEQ-T7	85726685700	42.16	93.08	93
Hi-C reads	MGISEQ-T7	79086390300	33.59	94.28	86

**Table 2:** Characteristics of ONT data for genome assembly.

Sequence	Platform	Total bases	GC content (%)	N50 (bp)	Sequence depth (×)
ONT reads	Nanopore	98574842275	29.55	36264	107

### Estimatie of genome size

**Fig. 1:**
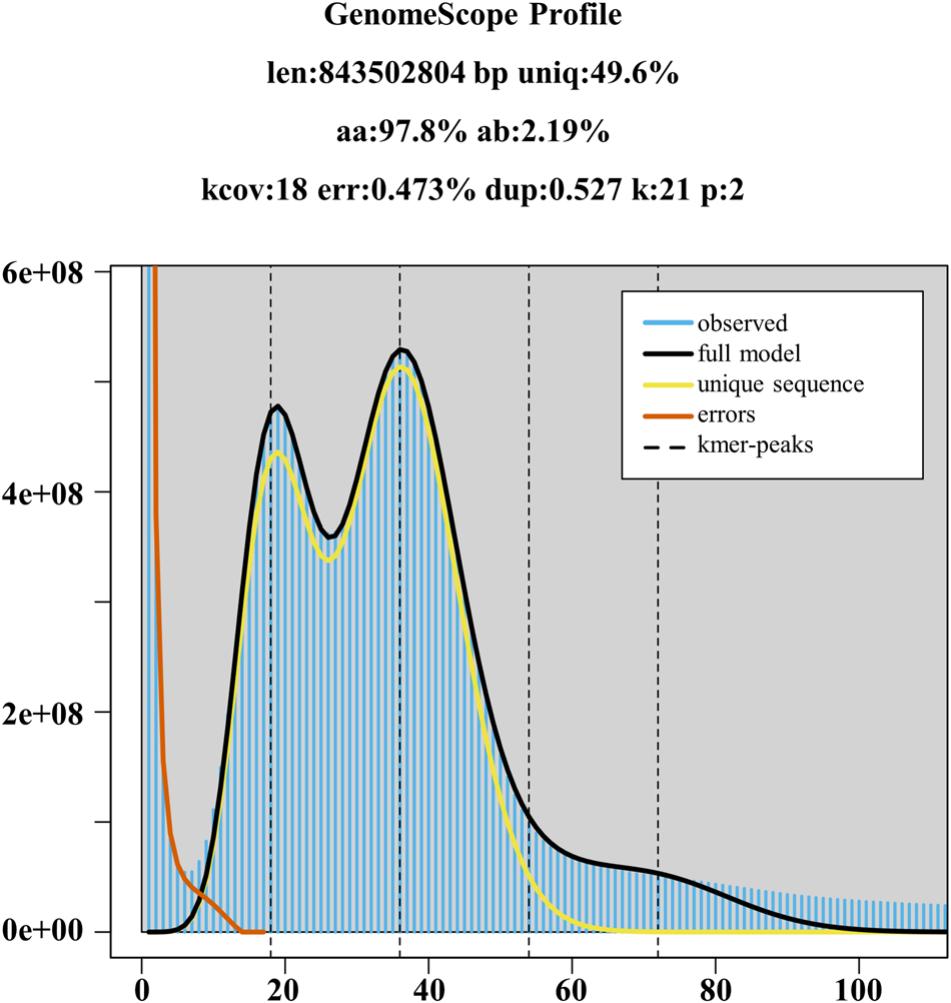
K-mer distribution (K = 21) of *Fructus hippophae* genome using GenomeScope 2.

Sequence adaptors, duplications, and low-quality reads from the original paired-end short DNA reads were filtered by Fastp^27^ with the parameters -n 0 -l 140. Then, 55 Gb of the clean reads from the MGI library were used to estimate the size, heterozygosity, and repeat content of the genome using Jellyfish^28^, with a 21-mer frequency and the parameter set as reads_cutoff = 1k, obetaining 47716688158 k-mer. Next, the Genomescope v2.0^29^ was used to analyze the K-mer frequency distribution. Ultimately, the genome size was estimated to be 843 Mb with 2.19% heterozygosity and 49.6% repetitive sequences (Fig. 1).

### Genome *de novo* assembly

**Fig. 2:**
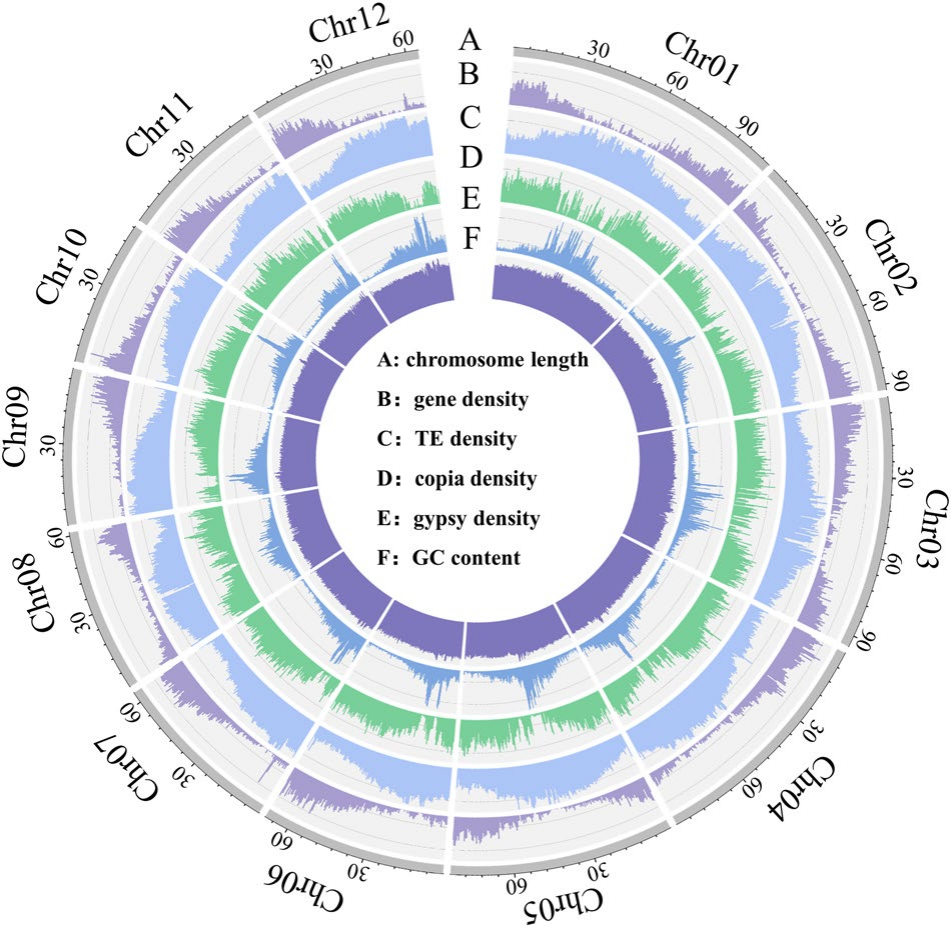
Circos plot of distribution of the *Fructus hippophae* genomic elements. The tracks indicate (**A**) length of chromosomes, (**B**) distribution of genes on different chromosomes, (**C**) distribution of transposable elements on different chromosomes, (**D**) distribution of copia elements on different chromosomes, (**E**) distribution of gypsy elements on different chromosomes, (**F**) GC content of different chromosomes. The densities of genes, TEs, copia elements, gypsy elements and GC were calculated in 500 kb windows.

**Fig. 3:**
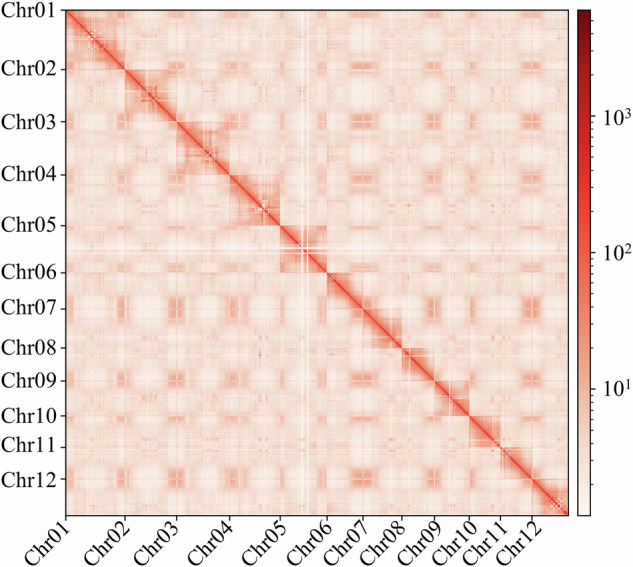
Heatmap of genome-wide Hi-C data of *Fructus hippophae* chromosomes. The frequency of Hi-C interaction links is represented by colors, ranges from orange (low) to dark red (high).

The genome was assembled by integrating the clean Nanorpore long reads, MGI short reads and Hi-C reads. First, *de novo* genome assembly was performed by NextDenovo (v2.5.0) (https://github.com/Nextomics/NextDenovo) with the high-quality ONT reads. Then, the clean NGS reads were used for four-rounds of self-correction and three-rounds of consensus correction by Nextpolish (v1.4.1)^30^ with the task parameter = best. Next, the redundant sequences resulting from heterozygosity were removed with the purge-dups (v1.2.5)^31^ pipeline. After assembly, a 921.69 Mb draft genome, including 723 contigs and the N50 reaching 14.8 Mb, was obtained (Table 3). Additionally, Hicpro (v3.1.0)^32^ was used to further validate the Hi-C reads, and 3D-DNA^33^ was then used to organize and anchor the contigs into draft chromosomes. Manual check and refinement to the cluster, order, and orientation of the draft assembly were carried out using Juicebox assembly tools^34^. Ultimately, the final genome was 918.59 Mb in size and consisted of 253 scaffolds with an N50 length up to 83.64 Mb, including 12 pseudochromosomes that accounted for 97.14% of the total genome length (Table 4). Circos plot of the distribution of the genomic elements (Fig. 2) was generated by shinyCircos v2.0 (https://venyao.xyz/shinyCircos/) and the heatmap of genome-wide Hi-C data (Fig. 3) of the *Fructus hippophae* genome chromosomes was drawn by hicexplorer.

**Table 3:** Characteristics of the *Fructus hippophae* genome at contig level.

Features	Statistics
Sequenced genome size (Mb)	921.69
Number of contigs	723
Contig N50 (bp)	14835755
Contig N90 (bp)	420505
Max contig size (bp)	45242531

**Table 4:** Characteristics of the *Fructus hippophae* genome at scaffold level.

Features	Statistics
Number of Chromosomes	12
Scaffold N50 (bp)	83648241
Scaffold N90 (bp)	55834877
GC content (%)	30
Max scoffold size (bp)	105172879
Total Size (Mb)	918.59

### Repetitive elements identification

The transposable elements (TEs) in the genome were identified and annotated by Extensive de-novo TE Annotator (EDTA) v2.1.2^35^ and classified by TEsorter (v1.3)^36^, DeepTE^37^, and LTR_FINDER^38^. Finally, 913550 bp of repeat elements were predicted, occupying 61.02% of the total genome length. The TEs could be classified into five categories after annotation, including long terminal repeats (LTR), tandem inverted repeats (TIR), non-LTR, non-TIR, and others. Of these, *Gypsy* occupied the highest proportion (35.65%) and was evenly distributed on 12 pseudochromosomes in the genome, followed by *Copia* with 19.81% occupation and high abundance in the central region of the genome (Table 5, Fig. 2).

**Table 5:** Summary of transposable elements in *Fructus hippophae* genome.

Type		count	masked (bp)	masked (%)
LTR	unknown	129541	66497775	7.24
	Gypsy	236098	159424221	17.36
	Copia	215332	155535260	16.94
TIR	DTA	85840	25710480	2.80
	DTH	28546	7676719	0.84
	DTM	78246	49437657	5.38
	DTC	88257	38449970	4.19
	DTT	38064	8859044	0.96
non-LTR	LINE_element	7529	3229551	0.35
	tRNA_SINE	1123	169924	0.02
	Penelope	1854	744765	0.08
	unknown	25098	23311951	2.54
non-TIR	helitron	63779	25541983	2.78
others	DNA_transposon	45599	11813872	1.29
	low_complexity	78	467391	0.05
	repeat_region	25227	7930950	0.86
Total		1070211	584801513	63.68

### Protein-coding genes prediction

Simultaneously, Repeatmasker (v4.1.2-p1)^39^ software was used for repeat masking. The masked genome was then subjected to gene prediction. First, structure annotation of the protein-coding genes was predicted using braker^40^ and tsebra^41^ software by integrating evidence from homology-, *de nove-* and transcriptome-based annotations. Maker (v3.01.04)^42^ and EVidenceModeler (v1.1.1) pipelines^43^ were used to integrate the evidence for non-redundant gene models, and the GFF3 file locating the gene, coding sequence, protein, and mRNA positions was obtained. Finally, a total of 36475 protein-coding genes were predicted, with gene lengths of 158 to 127368 bp. Additionally, 35943 (98.54%) of the predicted genes were allocated to the 12 chromosomes, and the gene distribution showed a higher density at the ends of the chromosomes.

### Genes function and non-coding RNA annotation

The functional annotations of the predicted genes were further annotated by homologous searches against public databases using BLASTP^44^ with the e-value cutoff = 1e-10, including NR, Swissprot^45^, Translated European Molecular Biology Laboratory (TrEMBL)^46^, KOG, GO^47^, KEGG^48^ and COG. Overall, 99.31% of the genes were functionally annotated. Among them 98.71%, 99.31%, 70.27%, 53.44%, 44.17%, 50.4%, and 37.83% gene were annotated in NR, TrEMBL^46^, Swissprot^45^, KOG, KEGG^48^, GO^47^ and COG^49^ databases, respectively (Table 6). Non-conding RNAs were identified using cmscan^50^ search against the RNA families database (Rfam)^51^ with default parameters. Finally, 10376 non-coding RNAs(1483600 bp), including 1041 transfer RNA (77105 bp), 750 ribosomal RNA (489313 bp), 84 spliceosomal nuclear RNA (11878 bp), 185 microRNA (23364 bp) and 9626 other types of RNA (858501 bp) were identified in *Fructus hippophae* (Table 7).

**Table 6:** Statistical analysis of the functional gene annotations of the *Fructus hippophae* genome.

Database	anno_num	ratio(%)
COG	13800	37.83
GO	18384	50.40
KEGG	16111	44.17
KOG	19494	53.44
Swissprot	25632	70.27
TrEMBL	36226	99.31
NR	36004	98.71
Total_annotated	36226	99.31

**Table 7:** Classification of non-coding RNA in the *Fructus hippophae* genome.

	Counts	Masked (bp)
miRNA	185	23364
tRNA	1041	77105
snRNA	245	23439
rRNA	750	489313
spliceosomal_RNA	84	11878
orthers	9626	858501
Total	10376	1483600

## Data Records

The genomic WGS sequencing data were deposited in the NCBI Sequence Read Archive (SRA) database under the BioProject PRJNA1003561.

The genomic NGS data were deposited in the SRA at NCBI SRR25591597^52^.

The genomic ONT data were deposited in the SRA at NCBI SRR25591606^53^ and SRR25591605^54^.

The RNA short reads of leaves and stems with 3 dupication were deposited in the SRA at NCBI SRR25591604^55^, SRR25591603^56^, SRR25591602^57^, SRR25591601^58^, SRR25591600^59^, SRR25591599^60^, SRR25591596^61^, SRR25591595^62^, SRR25591594^63^, SRR25591593^64^, SRR25591592^65^, and SRR25591591^66^.

Hi-C data were deposited in the SRA at NCBI SRR25591598^67^.

The final chromodome assembly and genome annotation files are available in GenBank^67^.

## Technical Validation

**Fig. 4:**
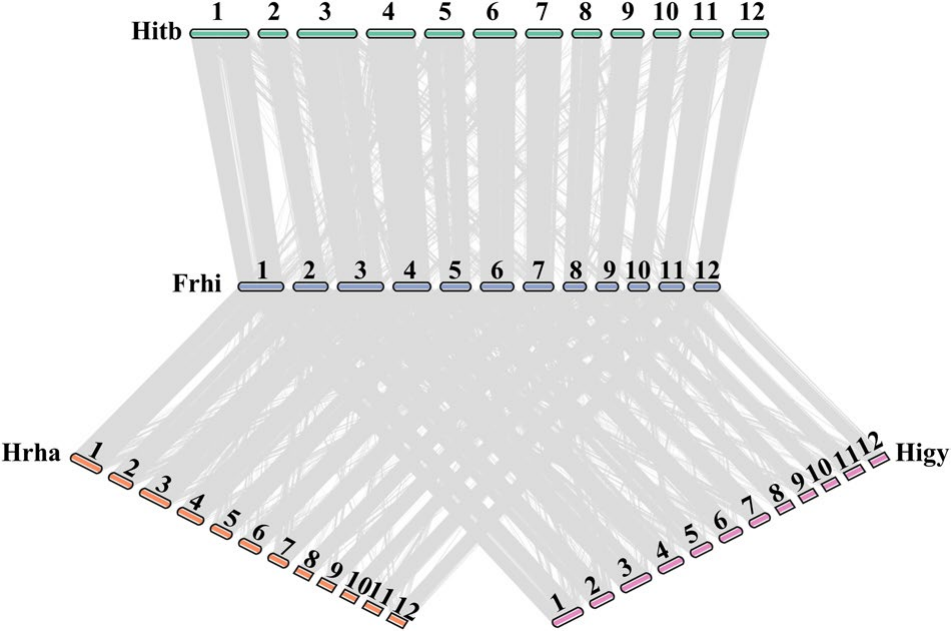
Genome synteny is observed among *F. hippophae* and three other Sea buckthorn species: Hrha for *Hippophae rhamnoides* ssp. *sinensis*, Frhi for Fructus hippophae, Hitb for *Hippophae tibetana*, and Higy for *Hippophae gyantsensis* genomes. Chromosome numbers 1–12 represent the chromosomes 1 through 12 of the four Sea buckthorn species.

Here, several strategies were taken to assess the genome quality. The completeness of the non-redundant draft genome was evaluated using Benchmarking Universal Single-Copy Orthologs (BUSCO)^68^ with the embryophyta odb10 dataset, which consists of 1614 single copy genes with the default parameters. Revealing that 98.8% of these genes exhibited complete coverage. Among them, 87.2% were complete and only 1% were missing (Table 8). Additionally, coverage was also estimated by mapping the NGS reads and ONT reads to the assembled genome with BWA-mem2 (v2.2) (https://github.com/bwa-mem2/bwa-mem2) and minimap2, respectively. The coverage was calculated by SAMtools^69^, indicating that 93.9% of the DNA short reads mapped to the assembled genome. Furthermore, the clean RNA reads were aligned back to the draft genome using HISAT2,with 99.96% of the uniquely mapped transcriptome reads suggesting comprehensive genome coverage. Given the existence of published sea buckthorn genomes, we also compared the gene structure between *F. hippophae* and other three sea buckthorn species using JCVI. Blocks with a span lower than 10 were filtered out, revealing a strong collinearity relationship (Fig. 4). In summary, the combined results from BUSCO, mapping coverage, and collinearity analysis demonstrate the high quality of our *F. hippophae* genome.

**Table 8:** Statistics for genome assessment using BUSCO.

BUSCO	%
Genome Complete Buscos	98.8
Complete and aingle-copy Buscos	87.2
Complete and duplicated Buscos	11.6
Fragemented Buscos	0.2
Missing Buscos	1.0

## Data Availability

No specific code was developed in this work.
